# The value revitalization model of Qajar religious schools in Tehran

**DOI:** 10.1186/s40494-022-00658-w

**Published:** 2022-02-19

**Authors:** Mohammad Sadegh Taher Tolou Del, Bahram Saleh Sedghpour, Sina Kamali Tabrizi

**Affiliations:** 1 Faculty of Architectural Engineering and Urban Design, Shahid Rajaee Teacher Training University, Tehran, Iran; 2 Faculty of Humanities, Shahid Rajaee Teacher Training University, Tehran, Iran; 3 Faculty of Architectural Engineering & Urban Design, Shahid Rajaee Teacher Training University, Tehran, Iran

**Keywords:** Value revitalization, Religious schools, Historical monuments, Qajar era, Virtual tour

## Abstract

Religious schools are institutions for teaching Islamic sciences. Nowadays, many religious schools in Tehran, which were built during the Qajar period, have been destroyed due to natural erosion and urban expansion. Since there is no comprehensive research on the conservation and revitalization of these schools, the present study aims to model the value revitalization of Qajar religious schools in Tehran. The present research is a mixed-methods study (a qualitative-quantitative study using a simulation). The data required are collected using a questionnaire. The statistical population includes school teachers who complete the structured questionnaire after visiting the schools through a virtual tour. The teachers are selected using a random sampling technique and the sample size (number of participated teachers) is 948. The sampling adequacy is confirmed with the KMO test. The reliability and validity of the questionnaire are also verified by Cronbach's alpha and the model fit index, respectively. The data are analyzed by modeling and path analysis in SPSS and AMOS software. Research results show that architectural phenomena (i.e., independent variable), through value conservation (i.e., mediator variable), have the most significant effect on the value revitalization (i.e., dependent variable) of Qajar religious schools in Tehran. Also, the path analysis shows three essential relations with a large effect size in the model: (1) The conservation of compound values influenced by social interactions in the building revitalizes the semi-tangible aesthetic factor of the building; (2) The conservation of physical values influenced by the building structure revitalizes the tangible aesthetic factor of the building; and (3) The conservation of semantic values influenced by the moral values of the building revitalizes the intangible aesthetic and educational factors of the building. In value revitalization, two basic aesthetic and educational factors play the most important role because they do not influence any other variable while all variables influence them.

## Introduction

Qajar religious schools are all located in the old grand bazaar of Tehran. Due to the high economic value of the land, most of these buildings have been destroyed and some shops or stores have been built in their places. Also, in some of the remaining schools, only spaces with less important uses have been demolished and some shops have been built in their place. About these schools, the biggest concern is their unprincipled and unresearched restoration, which has damaged their physical values. For example, they were restored regardless of their original materials or main ornamentations, making the restored building to be not in harmony with the original one and thereby damaging the authenticity of the building. Also, the most important semantic values in these buildings are the active presence of people to do religious affairs or teach religious lessons to students. Due to the ban on the use of schools by their owners, the intangible and semantic values of education and worship have diminished.

### Literature review

Various studies have been conducted in the field of Qajar religious schools in Tehran, including the history of education [1–4]; space analysis [5–7]; Architectural Typology [5, 8, 9], and spatial evolution [10–14].

The study by Taher-Toloudel et al. is the only research on the value conservation and revitalization of these schools. The results of their research have indicated that it is not adequate to pay attention only to the physical and tangible aspects of the building for the value conservation of the schools and it is required to consider the non-physical and intangible aspects of the building [15]. In another study, they have also identified seven factors effective in the value revitalization of these buildings based on interviews with experts. These seven factors include climatic architecture (climatic factor), resilient architecture (resilient factor), spiritual architecture (spiritual factor), environmental aesthetics (aesthetic factor), educational architecture (educational factor), structural architecture (structural factor), and site visiting (tourism factor) [16]. Moreover, according to the research method used, they have identified only the factors effective in the value revitalization and their constituent variables and the relationships between factors and variables have remained unknown. Therefore, the present study aims to investigate the relationships between factors and variables. The main research hypothesis is based on the fact that architectural phenomena, through value conservation, have the most significant effect on the value revitalization of Qajar religious schools in Tehran.

This article is the third part of an investigation into the value revitalization of Qajar religious schools in Tehran.

#### Research variables

**Fig. 1:**
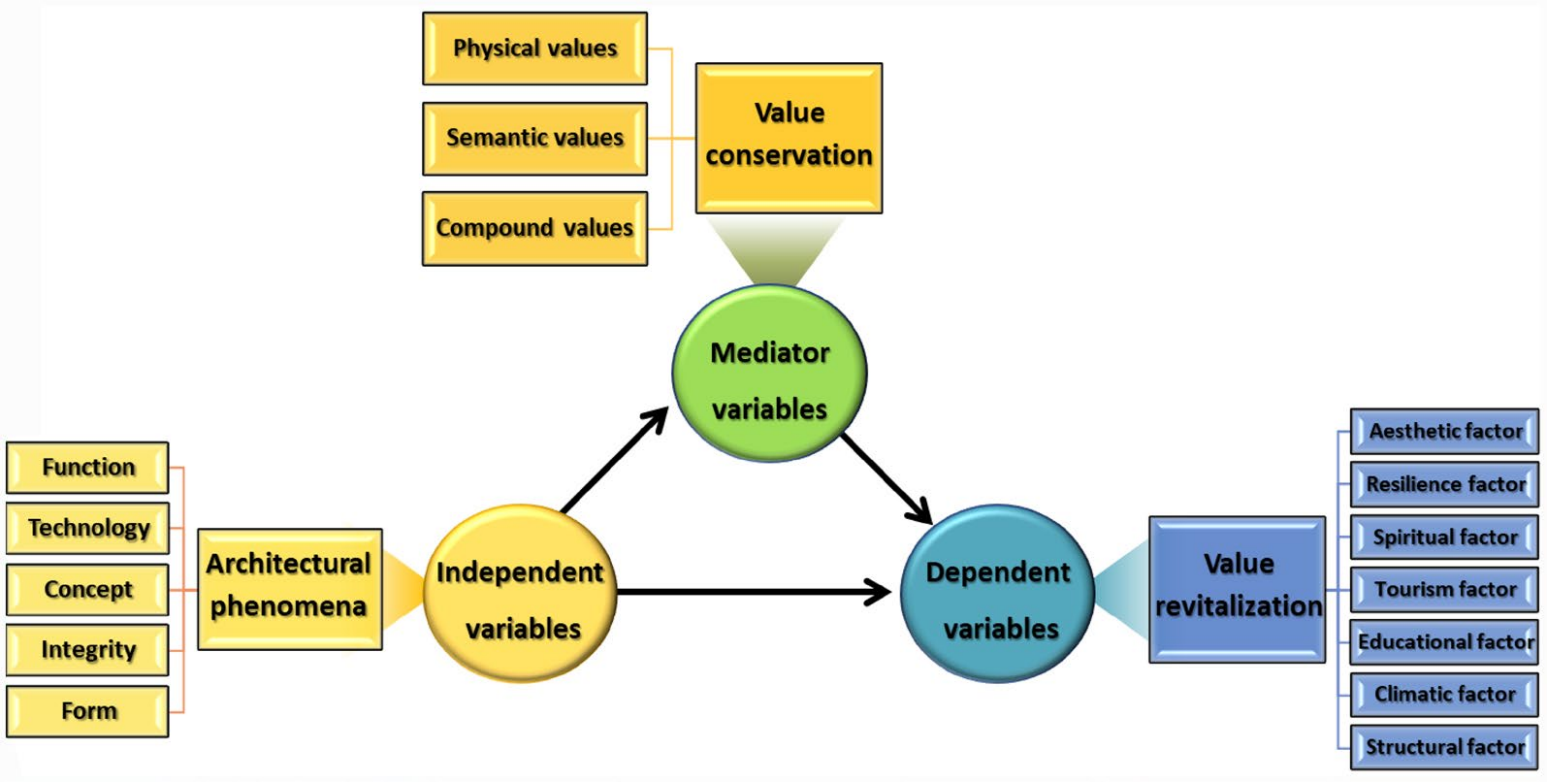
Research variables, (Source: Authors)

In the present study, the independent variable is architectural phenomena, the mediator variable is value conservation, and the dependent variable is value revitalization (Fig. 1).

Independent and mediator variables were extracted using the study results by [15] and dependent variables based on the results of the study by [16].

#### Independent variables: architectural phenomena

Architectural phenomena are classified into five classes of form, function, concept, technology, and integrity based on literature review and interviews with 25 architectural experts [15].

Form: The form refers to the shape and arrangement of components and the visible aspect of the architectural work [17]. It is the perceivable character, and identity of objects, and objects are known and distinguished with it. While the architectural form is a visible image of the material shaping it. Thus, it has components, proportions, and size. It includes and conveys the concept of human and the characteristics of the environment, and is a function of how humans perceive the world [18]. The form is the most main architectural factor and must be coordinated with factors such as value system, cultures, environmental, functional, as well as sustainable conditions [19]. The form refers to the appearance and three-dimensional shape of these schools.

Function: In the present study, function refers to the use provided for the users by the building. The functions of religious schools are including educational, residential, and devotional uses [15].

Concept: In architecture, a concept is actually an idea, a source of inspiration, or a motivational thought according to which actions are taken [20]. In fact, it is a relationship between subjectivity and objectivity, that is the architect uses the concept to represent his mental design by designing a building in reality [21]. The architecture was considered a symbolic language that could present spiritual concepts with patterns understandable to human beings. In religious schools, since architecture aims to capture the soul and intellect, geometry has become a tool for the Iranian architects to develop the forms of plants and animals that were inherently sacred [22]. Thhie concept is to use ideas in school architecture based on spiritual and moral subjects.

Technology: Technology is a set of construction skills. Buildings, from early shelters to modern complex buildings, have been associated with the constant presence of a type of construction system for resistance to gravitational forces, winds, earthquakes, etc., throughout the periods of technological evolution [23, 24]. Throughout history, humans have always had to build strong and safe buildings to protect themselves against destructive factors and forces and to identify and control the forces on the architectural structure in a reliable way [25]. The structural technology used in these schools is the traditional structures of Iran, namely arches and domes. This type of structure, while being static and reliable, is beautiful and is not separate from architecture.

Integrity: It refers to architectural variables that cannot be placed in a single form, function, concept, and technology category. These variables are multidimensional, such as cultural, historical, and aesthetic values.

#### Mediator variables: value conservation

From the contemporary view towards conservation, the major issue is to conserve values. It is no longer a product and is itself a conservation process [26]. This process is called value-based conservation. The measurement of values plays a key role in all measures related to architectural heritage as Feilden [27] states, the first step in the conservation process is to set a goal and then prioritize the existing values in the building to understand and convey the message of the work. In general, conservation activities take place when the object or location is valuable, and therefore making decisions on how to treat and intervene in the work follows this value [28].

The ICOMOS New Zealand Charter is one of the charters in the field of value-based conservation. In this charter, conservation aims to preserve the values of the architectural heritage [29]. Conservation of architectural heritage values can be observed in the three main aspects including the conservation of tangible values (physical conservation), conservation of intangible values (semantic conservation), and conservation of semi-tangible values (compound conservation), to which different priorities are allocated in different societies, according to their cultural and environmental contexts [30, 31].

#### Dependent variables: value revitalization

Each architectural work has a special value and a special cultural-historical place in its society according to its time, location, architectural technique, common models at the time of construction, use, and form [32]. The value changes over time as the building remains unused, as well as due to natural disasters and human hazards, the architectural work will be exposed to deterioration and destruction and this raises the need to conserve it [30, 31]. In most countries, one of the measures taken to conserve historical monuments is to revive them through the revitalization of existing values in the building [33, 34]. Factors affecting the value revitalization of Qajar religious schools in Tehran, based on interviews with experts and using the Delphi method, and Q factor analysis, include seven factors (climatic, resilience, spiritual, aesthetic, educational, structural, and tourism) [16].

## Methodology

The present study is mixed-method research (qualitative, quantitative, and simulation) which was carried out using an exploratory approach. Since the concepts and variables were specialized, the statistical population included school teachers. The samples were selected using a simple random sampling technique. In simple random sampling, each member of the statistical population has an equal probability of being chosen [35]. For this purpose, first, a call has been announced to the research community, which included1000 teachers in all schools in the country, by Shahid Rajaee Teacher Training University. Out of the research community, only 948 teachers agreed to participate in the study. All teachers had an equal opportunity to participate in the research. That's why sampling was random. The sampling adequacy was investigated by the Kaiser-Mayer-Olkin (KMO) measure. KMO is deduced through the following formula.

$$ {\text{KMO}} = \frac{{\mathop {\Sigma \Sigma }\nolimits_{i \ne j} \,r_{ij}^{2} }}{{ \mathop {\Sigma \Sigma }\nolimits_{i \ne j} \,r_{ij}^{2} + \mathop {\Sigma \Sigma }\nolimits_{i \ne j} \,a_{ij}^{2} }} $$

where rij is the simple correlation coefficient between variables, aij is the partial correlation coefficient between variables [36]. If its value is above 0.6, the sampling adequacy will be confirmed [37, 38]. According to the obtained KMO value (0.704), the sampling adequacy is confirmed (Table 1).

**Table 1:** KMO and Bartlett’s test

Kaiser–meyer–olkin measure of sampling adequacy		0.704
Bartlett's test of sphericity	Approx. Chi-Square	32,635.502
	df	105
	Sig.	0.000

The data required were collected using a questionnaire (Table 2). To measure the variables, a structured questionnaire including 92 questions was designed based on a 5-point Likert scale (1: Strongly disagree, 3: Neither agree nor disagree, and 5: Strongly agree) [39]. In this way, variables can be ranked and quantified [40, 41].

**Table 2:** In each question asked, to what extent do you agree with the sentences provided?

1	The geometric proportions of the schools are designed according to the climate of the region
2	The rooflines of the schools are designed according to the climate of the region
3	The shape of the school roofs is designed according to the climate of the region
4	School ornamentations are designed according to the climate of the area
5	The shape and form of the schools are designed according to the climate of the region
6	The identity values of the schools are considered according to the climate
7	The structures of the schools have been implemented according to the climate of the region
8	The arches and roofs of the schools have been implemented according to the climate of the region
9	The integrity of the schools is protected according to the climate of the area
10	The authenticity of the schools is protected according to the climate of the region
11	The architecture of the schools is designed according to the climate of the region
12	The shape of the school roofs has been implemented according to the climate of the region
13	School ornamentations are considered according to the climate of the region
14	The geometric proportions of the schools have been influential in the sustainability of their buildings
15	The rooflines of the schools have been influential in the sustainability of their buildings
16	The design of the school plans has been influential in the sustainability of their buildings
17	School facades have been influential in the sustainability and durability of their buildings
18	The shape and form of the schools have been influential in the sustainability of their buildings
19	The appearance of the schools has been influential in the sustainability of their buildings
20	Controlling temperature and humidity in the schools has been done according to the sustainability of their buildings
21	The lighting of the schools has been influential in the sustainability of their buildings
22	The identity values of the schools are considered according to the sustainability of their buildings
23	The symbolism of the schools has been influential in the sustainability of this building
24	The grandeur of the schools has been influential in the sustainability of their buildings
25	The ability of the schools to resile has been influential in the sustainability of their buildings
26	The structures of the schools have been implemented according to the sustainability of their buildings
27	The pillars of the schools have been constructed according to the sustainability of their buildings
28	The arches and roofs of the schools have been implemented according to the sustainability of their buildings
29	The cultural values of the schools are considered according to the sustainability of their buildings
30	The locations of the schools have been influential in the sustainability of their buildings
31	The aesthetic values of the schools have been influential in the sustainability of their buildings
32	The authenticity of the schools has been influential in the sustainability of their buildings
33	The architecture of the schools has been influential in the sustainability of their buildings
34	The sense of belonging to the schools has been influential in the sustainability of their buildings
35	The schools' compatibility with the surrounding environment has been influential in the sustainability of their buildings
36	The artistic values of the schools have been influential in the sustainability of their buildings
37	The symbolism of the schools is considered according to the sustainability of their buildings
38	The grandeur of the schools is considered according to the sustainability of their buildings
39	The locations of the schools are considered according to the sustainability of their buildings
40	The aesthetic values of the schools are considered according to the sustainability of their buildings
41	The sense of belonging to the schools is considered according to the sustainability of their buildings
42	The geometric proportions of the schools are designed according to spiritual concepts
43	School facades are designed according to spiritual concepts
44	The shape and form of the schools are designed according to spiritual concepts
45	The identity values of the schools are considered according to spiritual concepts
46	The religious values of the schools are considered in terms of spiritual concepts
47	The cultural values of the schools are considered in terms of spiritual concepts
48	The historical values of the schools are protected under spiritual concepts
49	The integrity of the schools is protected under spiritual concepts
50	The authenticity of the schools is protected under spiritual concepts
51	School architecture is designed according to spiritual concepts
52	The aesthetic values of the schools are considered in terms of spiritual concepts
53	The geometric proportions of the schools are designed to show the beautiful environment
54	The landscapes of the schools are designed to show the beautiful environment
55	The schoolyards are designed to show the beautiful environment
56	The identity values of the schools are considered to show the beautiful environment
57	The aesthetic values of the schools are designed to show the beautiful environment
58	The architecture of the schools is designed to show the beautiful environment
59	The sense of belonging to the schools has been due to the beauty of the environment
60	The artistic values of the schools are designed to show the beautiful environment
61	The aesthetic values of the schools are considered to show the beautiful environment
62	The sense of belonging to the schools is considered according to the beauty of the environment
63	School ornamentations are designed according to educational concepts
64	The landscapes of the schools are designed according to educational concepts
65	The lighting of the schools has been influential in the quality of student education
66	The identity values of the schools are considered according to educational concepts
67	The educational space in the schools is designed according to educational concepts
68	The cultural values of the schools are considered in terms of the education of students
69	The locations of the schools have been influential in the education of students
70	The authenticity of the schools is protected according to educational concepts
71	School architecture is designed according to educational concepts
72	The possibility of social interactions in the schools is considered according to educational concepts
73	The sense of belonging to the schools has been influential in the education of students
74	School ornamentations are considered according to educational concepts
75	The locations of the schools are considered according to the education of students
76	The sense of belonging to the schools is considered according to educational concepts
77	School ornamentations are designed according to their building structures
78	The amount of income of the schools has been influential in protecting their building structures
79	The identity values of the schools are considered according to their building structures
80	The strength of the schools has depended on their building structures
81	The historical values of the schools are protected due to their building structures
82	School architecture is designed according to the building structure
83	School ornamentations are designed according to the public visit
84	The windows and vents of the schools are designed according to the public visit
85	The schoolyard is designed according to the public visit
86	The arches and ceilings of the schools have been implemented according to the public visit
87	The historical values of the schools are protected under public visits
88	The integrity of the schools is protected under public visits
89	The authenticity of the schools is protected due to the public visit
90	The globalization of the schools is influential in showing the public visit
91	School ornamentations are considered according to the public visit
92	The locations of the schools are considered according to the public visit

The questions were designed based on the Table of Specifications (TOS) (Table 3). This table is a reliable tool for evaluating the variables that the test intends to measure [42]. The TOS includes independent variables in columns, and mediator and dependent variables in rows. The questions were designed based on the intersection points in the matrix to allow evaluating the relationships between variables.

**Table 3:** The table of specifications

Content	Goals											
	Climatic factor			Resilience factor			Spiritual factor			Aesthetic factor		
	Physical values	Semantic values	Compound values	Physical values	Semantic values	Compound values	Physical values	Semantic values	Compound values	Physical values	Semantic values	Compound values
Form												
Building proportions	–	–	1	–	–	14	–	–	42	–	–	53
Skyline	–	–	2	–	–	15	–	–	–	–	–	–
Plan layout	–	–	–	–	–	16	–	–	–	–	–	–
Roofing and covering	12	–	3	–	–	–	–	–	–	–	–	–
Facades design	–	–	–	–	–	17	–	–	43	–	–	–
Architectural ornamentation	–	13	4	–	–	–	–	–	–	–	–	–
Physical form	–	–	5	–	–	18	–	–	44	–	–	–
Landscape value	–	–	–	–	–	19	–	–	–	–	–	54
Function												
Windows and vents	–	–	–	–	–	–	–	–	–	–	–	–
Yard and campus	–	–	–	–	–	–	–	–	–	–	–	55
Temperature and humidity	–	–	–	20	–	–	–	–	–	–	–	–
Lighting	–	–	–	–	–	21	–	–	–	–	–	–
Economic value	–	–	–	–	–	–	–	–	–	–	–	–
Concept												
Identity value	–	6	–	–	22	–	–	45	–	–	56	–
Spiritual value	–	–	–	–	–	–	–	46	–	–	–	–
Symbolic value	–	–	–	–	37	23	–	–	–	–	–	–
Grandeur value	–	–	–	–	38	24	–	–	–	–	–	–
Resilience value	–	–	–	–	–	25	–	–	–	–	–	–
Technology												
Structural system	7	–	–	26	–	–	–	–	–	–	–	–
Columns and bases	–	–	–	27	–	–	–	–	–	–	–	–
Vault and ceiling	8	–	–	28	–	–	–	–	–	–	–	–
Scientific value	–	–	–	–	–	–	–	–	–	–	–	–
Integrity												
Cultural value	–	–	–	–	29	–	–	47	–	–	–	–
Situational value	–	–	–	–	39	30	–	–	–	–	–	–
Historical value	–	–	–	–	–	–	–	–	48	–	–	–
Aesthetic value	–	–	–	–	40	31	–	52	–	–	61	57
Integrity value	–	–	9	–	–	–	–	–	49	–	–	–
Authenticity	–	–	10	–	–	32	–	–	50	–	–	–
Architectural value	–	–	11	–	–	33	–	–	51	–	–	58
Global registration value	–	–	–	–	–	–	–	–	–	–	–	–
Social value	–	–	–	–	–	–	–	–	–	–	–	–
Sense of belonging value	–	–	–	–	41	34	–	–	–	–	62	59
Compatibility value	–	–	–	–	–	35	–	–	–	–	–	–
Artistic value	–	–	–	–	–	36	–	–	–	–	–	60

Content	Goals								
	Educational factor			Structural factor			Tourism factor		
	Physical values	Semantic values	Compound values	Physical values	Semantic values	Compound values	Physical values	Semantic values	Compound values
Form									
Building proportions	–	–	–	–	–	–	–	–	–
Skyline	–	–	–	–	–	–	–	–	–
Plan layout	–	–	–	–	–	–	–	–	–
Roofing and covering	–	–	–	–	–	–	–	–	–
Facades design	–	–	–	–	–	–	–	–	–
Architectural ornamentation	–	74	63	–	–	77	–	91	83
Physical form	–	–	–	–	–	–	–	–	–
Landscape value	–	–	64	–	–	–	–	–	–
Function									
Windows and vents	–	–	–	–	–	–	–	–	84
Yard and campus	–	–	–	–	–	–	–	–	85
Temperature and humidity	–	–	–	–	–	–	–	–	–
Lighting	–	–	65	–	–	–	–	–	–
Economic value	–	–	–	–	–	78	–	–	–
Concept									
Identity value	–	66	–	–	79	–	–	–	–
Spiritual value	–	–	–	–	–	–	–	–	–
Symbolic value	–	–	–	–	–	–	–	–	–
Grandeur value	–	–	–	–	–	–	–	–	–
Resilience value	–	–	–	–	–	–	–	–	–
Technology									
Structural system	–	–	–	80	–	–	–	–	–
Columns and bases	–	–	–	–	–	–	–	–	–
Vault and ceiling	–	–	–	–	–	–	86	–	–
Scientific value	–	–	67	–	–	–	–	–	–
Integrity									
Cultural value	–	68	–	–	–	–	–	–	–
Situational value	–	75	69	–	–	–	–	92	–
Historical value	–	–	–	–	–	81	–	–	87
Aesthetic value	–	–	–	–	–	–	–	–	–
Integrity value	–	–	–	–	–	–	–	–	88
Authenticity	–	–	70	–	–	–	–	–	89
Architectural value	–	–	71	–	–	82	–	–	–
Global registration value	–	–	–	–	–	–	–	–	90
Social value	–	72	–	–	–	–	–	–	–
Sense of belonging value	–	76	73	–	–	–	–	–	–
Compatibility value	–	–	–	–	–	–	–	–	–
Artistic value	–	–	–	–	–	–	–	–	–

Out of 38 Qajar schools built in Tehran, 19 schools have been destroyed and cannot be studied. Out of 19 remaining schools, only for 12 schools, the authors were allowed to conduct a field study, as presented in Table 4.

**Table 4:** Case studies: Qajar religious schools in Tehran, (Source: Sina Kamali Tabrizi)

No.	School name	
1	The new Sepahsalar (Shahid Motahari)	
2	The old Sepahsalar (Shahid Beheshti)	
3	Philsof al-Dowleh	
4	Sadr	
5	Aqsa (Moshir al-Saltanah)	
6	Khan Marvi	
7	Memarbashi	
8	Haj Ghanbar Ali Khan	
9	Moayer Al-Mamalek	
10	Sheikh Abdol Hossein	
11	Khazen al-Molk	
12	Kazemieh	

All the schools were first photographed by Theta V 360-degree camera and their virtual tours were made separately using the 3DVista software (www.3dvista.com) and are available online on www.vrheritage.ir. Teachers were asked to first carefully review the virtual tours of all schools and then complete the questionnaire on the same website. The reliability of the data obtained in the study was investigated by Cronbach’s alpha. If Cronbach's alpha value is greater than 0.7, the research data will have desirable reliability [43]. Given that Cronbach's alpha value is estimated as 0.945, the reliability of the data obtained is confirmed. Cronbach’s alpha measures the internal consistency or reliability between several items. In other words, it estimates how reliable are the responses of a questionnaire by subjects, indicating the stability of the tools. However, Keith S. Taber [44] has reviewed 69 articles and stated that Cronbach’s alpha alone is not enough in some cases, and additional statistics can be provided. Structural equation modeling (SEM) reliability coefficients are often recommended as an alternative to Cronbach’s alpha [45]. Therefore, in addition to Cronbach's alpha, the results of the structural model fit in Table 5 confirm the reliability of the research.

**Table 5:** Descriptive information of model fit index, (Source: Authors)

Model	NPAR	CMIN	DF	P	CMIN/DF	GFI	NFI Delta1	CFI	RMSEA	PCLOSE
Default model	104	7.135	16	0.971	0.446	0.999	1.000	1.000	0.000	1.000

Using SPSS and AMOS software, the relationships among independent (architectural phenomena), mediator (value conservation), and dependent (value revitalization) variables were explained based on path analysis. AMOS is a visual program for structural equation modeling (SEM). Path analysis is a statistical method for applying the standardized regression coefficients (beta coefficients) in structural models. Path analysis aims to obtain quantitative estimates for causal relationships between a set of variables [46]. The analysis indicates the direction and intensity of the relationship between study variables [47]. The values indicating the direction and intensity of the relationships between the variables are called "path coefficients". Path coefficients are the same standardized regression coefficients. Therefore, simple linear regression should be used for path analysis [48]. Path analysis is an advanced statistical method that can be used to identify the indirect effects of each independent variable on the dependent variable, in addition to their direct effects [49]. Therefore, the most important advantage of path analysis over the regression analysis is that it allows identifying the indirect effects of each independent variable on the dependent variable, in addition to their direct effects while regression analysis only identifies the direct effect of each variable on the dependent variable [50]. So, in path analysis, there are several standardized regression line equations while there is only one standardized regression line equation in regression analysis [51].

Since the P-value (> 0.05), the CMIN / DF value (˂2), the GFI value (> 0.9), the NFI and CFI values (> 0.9), the RMSEA value (˂0.1), and the PCLOSE value (> 0.9) are within the acceptable ranges [52], the model has an acceptable fit index (Table 5).

## Results

The essential relations based on each factor's magnitude of correlation coefficients are identified and presented in Table 6.

**Table 6:** The essential relations based on Pearson's correlation coefficients

Main factor	Correlation coefficient	Assessed item
Tourism factor	0.715	The locations of the schools are considered according to the public visit
	0.661	The schoolyards are designed according to the public visit
Climatic factor	0.723	The arches and roofs of the schools have been implemented according to the climate of the region
	0.665	School ornamentations are designed according to the climate of the area
	0.637	The integrity of the schools is protected according to the climate of the area
	0.603	The shape of the school roofs has been implemented according to the climate of the region
Resilience factor	0.744	The locations of the schools are considered according to the stability of their buildings
	0.688	The cultural values of the schools are considered according to the sustainability of their buildings
	0.634	The appearance of the schools has been influential in the sustainability of their buildings
	0.625	The artistic values of the schools have been influential in the sustainability of their buildings
	0.629	The schools' compatibility with the surrounding environment has been influential in the sustainability of their buildings
	0.619	The identity values of the schools are considered according to the sustainability of their buildings
	0.614	School facades have been influential in the sustainability and durability of their buildings
	0.604	The shape and form of the schools have been influential in the sustainability of their buildings
Aesthetic factor	0.673	The sense of belonging to the schools has been due to the beauty of the environment
	0.645	The schoolyards are designed to show the beautiful environment
	0.635	The artistic values of the schools are designed to show the beautiful environment
	0.613	The landscapes of the schools are designed to show the beautiful environment
Spiritual factor	0.741	The cultural values of the schools are considered in terms of spiritual concepts
	0.734	The religious values of the schools are considered in terms of spiritual concepts
	0.632	The historical values of the schools are protected under spiritual concepts
Structural factor	0.636	School architecture is designed according to the building structure
Educational factor	0.741	The lighting of the schools has been influential in the quality of student education
	0.634	The locations of the schools have been influential in the education of students

Table 7 presents the results of path analysis, indicating the relation directions and path coefficients. In the table, one can see three types of effects among the variables, i.e., the direct, indirect, and total effects that variables have on each other.

**Table 7:** Direct, indirect, and total effects of variables based on the relations in the model (Source: Authors)

Affected variables	Relation direction	Effective variable	Direct effect	Indirect effect	Total effect
Mediator variables					
Semantic values	←	Concept	0.756^a^	0	0.756
Semantic values	←	Function	0.234^a^	0	0.234
Compound values	←	Integrity	0.737^a^	0	0.737
Physical values	←	Technology	0.837^a^	0.018	0.855
Dependent variables					
Tourism factor	←	Compound values	0.650^a^	0	0.650
Tourism factor	←	Function	0.276^a^	− 0.003	0.273
Spiritual factor	←	Semantic values	0.763^a^	0	0.763
Resilience factor	←	Compound values	0.644^a^	− 0.316	0.328
Resilience factor	←	Semantic values	1.043^a^	− 0.357	0.686
Educational factor	←	Semantic values	0.895^a^	0.045	0.940
Climatic factor	←	Compound values	1.056^a^	− 0.545	0.511
Climatic factor	←	Semantic values	0.713^a^	− 1.274	− 0.561
Climatic factor	←	Spiritual factor	− 0.646^a^	0.148	− 0.498
Climatic factor	←	Form	0.620^a^	0.228	0.848
Climatic factor	←	Physical values	0.330^a^	0.021	0.351
Aesthetic factor	←	Compound values	3.409^a^	− 1.614	1.795
Aesthetic factor	←	Semantic values	1.850^a^	− 2.932	− 1.082
Aesthetic factor	←	Tourism factor	− 0.808^a^	0.833	0.025
Aesthetic factor	←	Spiritual factor	− 1.209^a^	0.894	− 0.315
Aesthetic factor	←	Resilience factor	− 1.566^a^	0.527	− 1.039
Aesthetic factor	←	Climatic factor	− 0.927^a^	0.007	− 0.920
Aesthetic factor	←	Physical values	0.346^a^	− 0.280	0.066
Aesthetic factor	←	Structural factor	− 0.233^a^	0.188	− 0.045
Structural factor	←	Compound values	0.520^a^	0.086	0.606

^a^Significant at the level of 99% confidence

**Fig. 2:**
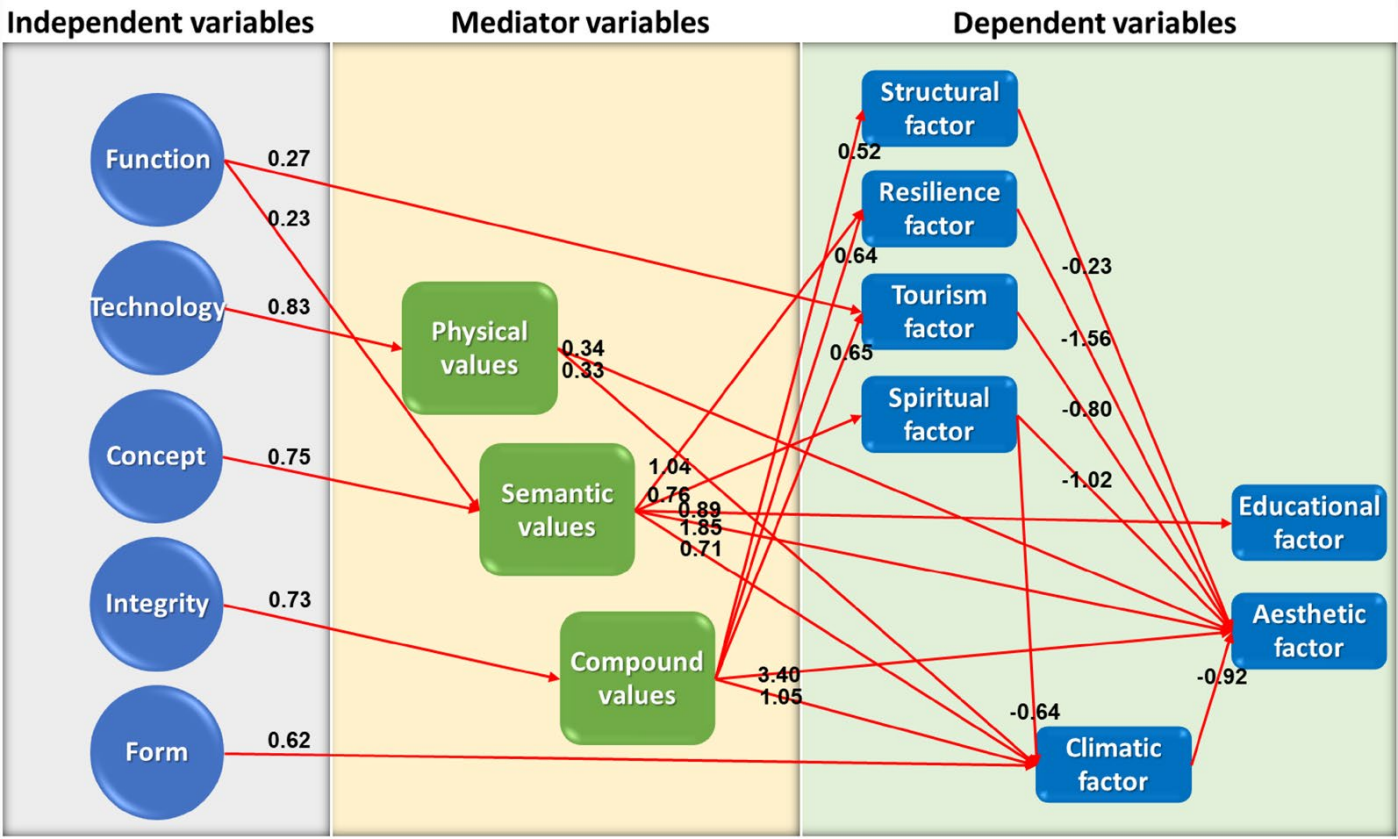
The value revitalization model of Qajar religious schools in Tehran (Source: Authors)

According to Table 7 and Fig. 2, regarding the direct effects of independent variables on the mediator ones, the greatest effect sizes are observed in the relations between "integrity and compound values", "technology and physical values", and "concept and semantic values". Moreover, the only independent variable that has a significant direct effect on the dependent variable is the "form" variable, which directly affects the "climatic factor" variable.

Regarding the direct effects of mediator variables on dependent ones, the greatest effect sizes are observed in the relations between the "compound values" and four variables of resilience, climatic, aesthetic, and tourism factors. Also, semantic values have the greatest effects on five variables of resilience, climatic, aesthetic, spiritual, and educational factors.

The dependent variables of resilience, climatic, tourism, and spiritual factors also have direct effects on the aesthetic factor. Moreover, the spiritual factor has a significant effect on the climatic factor. It should be noted that the aesthetic and educational factors are the most important dependent variables because they are only affected by other variables and do not affect any variables.

## Discussion

**Fig. 3:**
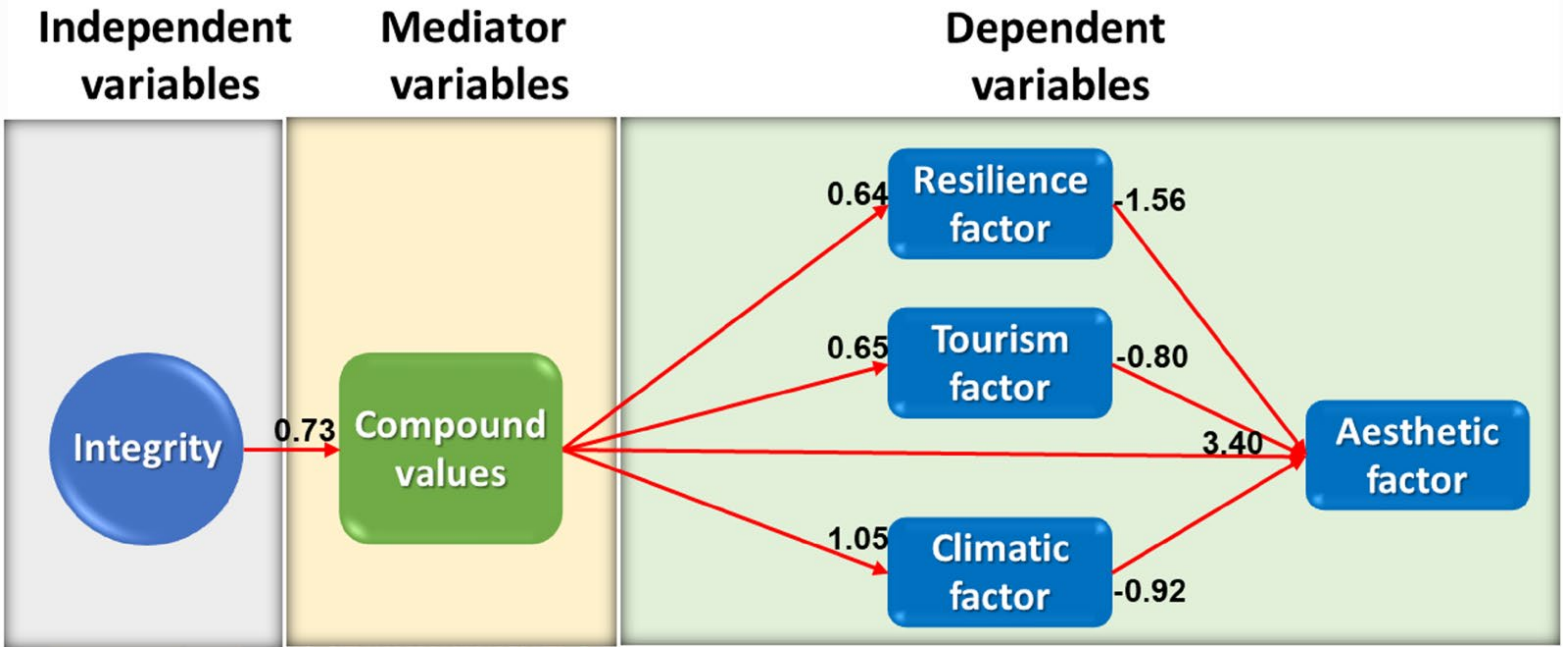
The effect of integrity on the aesthetic factor through compound values, (Source: Authors)

The discussion is based on path analysis for further interpretation of the results. Three main paths are identified and presented in the value revitalization model. In the analysis of the first path, the "integrity" variable has the most significant effect on the aesthetic factor through the compound values (Fig. 3). Also, resilience, tourism, and climatic factors act as catalysts in the relationship between compound values and the aesthetic factor. Social and endowment values are the most important sub-variables of the "integrity" variable. In other words, the presence of deeds of endowment and social interactions in the building has led to the conservation of the building and considering the active presence of tourists in the building, climatic, cultural, identity, and structural conditions, which are the most important variables of resilience, result in the revitalization of the building.

To confirm this issue, results indicated that the active participation of the people in the building has revived the social, cultural, and identity values in the building, leading to the conservation of the building. Also, those schools with the deeds of endowment have been less destructed and damaged than those with no deed of endowment. To confirm the relationship between social values and the tourism factor, Henderson's research indicated that due to the increased importance of tourism in the present era, the reuse of historical monuments and the active presence of tourists in them have made it possible to prevent a large number of monuments from being destructed and revitalize them through tourism [53].

**Fig. 4:**
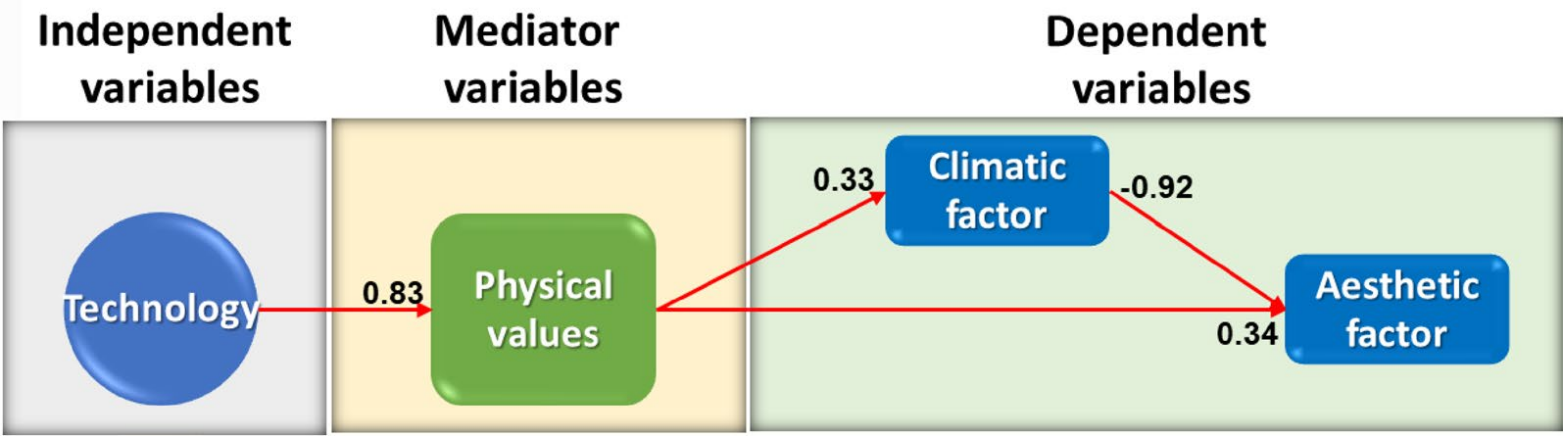
The effect of technology on the aesthetic factor through physical values, (Source: Authors)

In the analysis of the second path, the "technology" variable has the most significant effect on the aesthetic factor through physical values (Fig. 4). The climatic factor also acts as a catalyst in the relationship between physical values and the aesthetic factor. Vault and ceiling, scientific value, columns, and bases are the most important sub-variables of the "technology" variable. In other words, the structural elements affect the aesthetic aspect of the building through its physical conditions. The architecture of these buildings is such that body, structure, and beauty are not separate from each other. Here, beauty refers to the technical aspect that can be examined in the building mass, facades, forms, elements, and building design. Sabri, in her research, has also shown that vaults have various characteristics in terms of form, construction technique, and materials and these characteristics are very effective in identifying the identity signs and beauty of historical monuments [54].

**Fig. 5:**
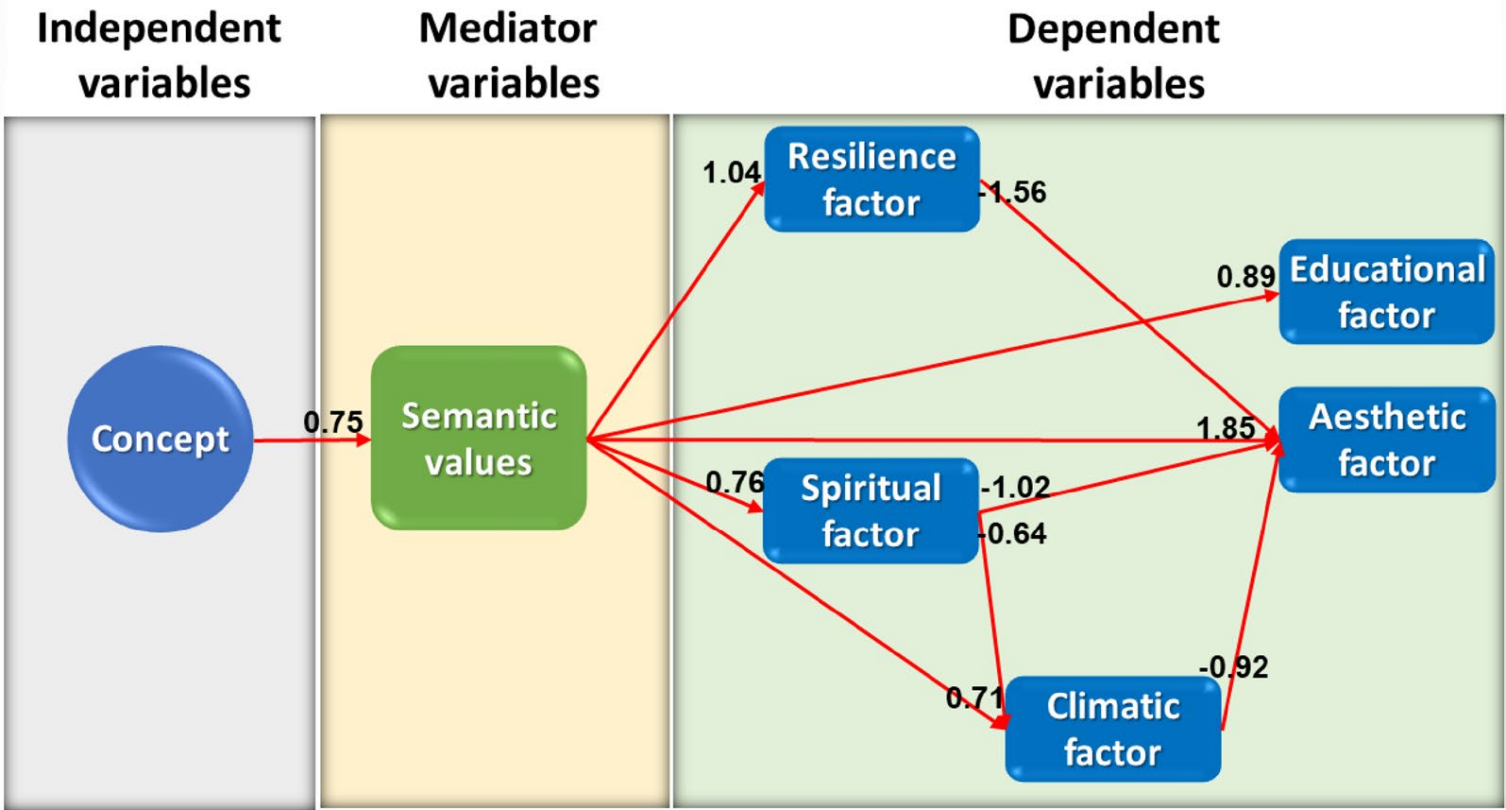
The effect of the "concept" variable on aesthetic and educational factors through semantic values (Source: Authors)

In the analysis of the third path, the "concept" variable has the greatest effect on the aesthetic and educational factors through semantic values (Fig. 5). Moreover, resilience, spiritual, and climatic factors act as catalysts in the relationship between semantic values and the aesthetic factor. The moral value is the most important sub-variable of the "concept" variable. In other words, those moral values creating meaning in the environment must be considered in beautifying spaces and designing educational spaces. In this regard, Karlstrom has stated that in the revitalization of historical monuments, contrary to Western views, maintaining the semantic and conceptual values of the building are more important and is more valuable than maintaining the original form of the building [55]. Throsby has acknowledged that any building reflecting a manifestation of a nation's religious and cultural beliefs has spiritual and semantic values and helps to strengthen the identity of society as a whole [56]. To confirm the relationship between the moral value and aesthetic and educational factors, Schiller and Snell have believed that it is only art and beauty through which a person can be hopefully moralized and a moral sense is brought him [57]. Thus, it is observed that dealing with art is a kind of moral education and a stage of it. It should be noted that when it comes to aesthetic education, we do not mean traditional education, which comes to mind with a specific curriculum. It means aesthetics and dealing with art that can have a positive effect on human beings. Therefore, although this type of education may not teach us scientific concepts, it makes individuals sensitive to some great human concepts, and the human being changes tangibly and morally through it.

## Conclusion

In this research, a methodology was presented. It can be helpful for revitalizing other architectural heritage. According to this methodology, considering the function of the building, the influential variables in the conservation process should be identified by researchers, using literature review, field investigation, interviews with experts, and building users. Then, the data obtained can be analyzed using quantitative methods to determine the relationships between variables. The identified variables and recognized relationships will be the basis of protective measures. According to the obtained results, the main research hypothesis stating "architectural phenomena have the most significant effect on the value revitalization of Qajar religious-educational buildings in Tehran through value conservation" is confirmed. According to the developed model, among the independent variables, only the "form" variable has a significant direct effect on the climatic factor (a dependent variable). Other independent variables, including concept, function, technology, and integrity, affect dependent variables through mediator variables. The path analysis shows that there are three significant relationships with a great effect size in the model: (1) The conservation of compound values influenced by social interactions in the building revitalizes the semi-tangible aesthetic factor of the building; (2) The conservation of physical values influenced by the building structure revitalizes the tangible aesthetic factor of the building; and (3) The conservation of semantic values influenced by the moral values of the building revitalizes the intangible aesthetic and educational factors of the building. In fact, it can be said that in value revitalization, only two basic aesthetic and educational factors play the most important role because they do not influence any other variables while all variables influence them. The educational factor (educational architecture) mainly aims to educate human beings to achieve excellence and perfection. From an architectural point of view, the context of this education is provided through the language of architecture, and based on the influence of the environment on the unconscious mind of individuals. Thus, the used architecture and concepts play a key role in educating people based on moral values and should be considered in the process of value revitalization. Moreover, most of the relationships in the model end in the aesthetic factor (environmental aesthetics). In the Qajar religious-educational buildings in Tehran, three compound, physical, and semantic values and five structural, tourism, spiritual, resilience, and climatic factors are dependent on the aesthetic factor. The aesthetic factor is important because the beauty that these buildings have brought to society is the reason for the survival of most of these buildings and it also represents the cultural and identity values of a society. That is why the public has cared about such buildings from the past to the present.

### Limitations

Due to the prevalence of coronavirus disease, it was not possible for the participants to visit the Qajar religious schools in Tehran; instead, a virtual tour was held. Moreover, since many Qajar religious-educational buildings in Tehran were demolished, authors could not access them and, to make matter worse, historical documentation on them is scarce.

### Suggestions

According to the ICOMOS New Zealand Charter of 2010, the functional use of the building can be changed if the building values are not damaged. If some of these schools are not be revitalized with their original functions, by changing their functions based on two aesthetic and educational factors, such as the Faculty of Art and Architecture, some necessary measures should be taken to reuse the buildings. It is also suggested to make policy at the macro level to make it possible for the public to visit these buildings in the form of tourism. Finally, it is suggested to separately develop a conservation plan for each Qajar religious school in Tehran considering the physical-semantic conditions of each school according to the value revitalization model presented in this study.

## Data Availability

All the data generated or analyzed during this study are included in this published paper.
